# Implementing Integrative Psychosocial Care for Siblings and Caregivers of Youth with Cancer

**DOI:** 10.3390/children12101335

**Published:** 2025-10-04

**Authors:** Joanna Patten, Helena Hillinga Haas, Riley Coyle, David Knott

**Affiliations:** 1 Seattle Children’s Hospital, Seattle, WA 98105, USA; riley.coyle@seattlechildrens.org (R.C.); david.knott@seattlechildrens.org (D.K.); 2 Dream Big Wellness, Seattle, WA 98125, USA; helena@dreambigwellness.org

**Keywords:** art therapy, integrative care, child life, sibling, caregiver, cancer, psychosocial

## Abstract

**Highlights:**

**What are the main findings?**

- Integrative care for siblings and caregivers of youth with cancer can be developed and implemented with interdisciplinary collaboration.
- Short-term funding can be leveraged to support development and implementation of integrative care for siblings and caregivers of youth with cancer.

**What is the implication of the main finding?**

- Demonstrating utilization of and positive family feedback for integrative psychosocial care for siblings and caregivers of youth with cancer may support sustained operational funding of services for these important yet often underserved family members.

**Abstract:**

Background/Objectives: Psychosocial care for siblings and caregivers of youth with cancer (SCYC) is a critical yet under-implemented component of comprehensive pediatric oncology care, as outlined by the Standards for Psychosocial Care for Children with Cancer and Their Families. Despite evidence supporting psychosocial interventions, such as integrative care interventions, as effective for stress mitigation and coping, barriers to implementation include revenue-generating funding models and siloed psychosocial disciplines, which hinder accessibility for adult caregivers within pediatric institutions and geographically dispersed families. This manuscript describes the relevant extant literature as well as a model for leveraging short-term funding opportunities and interdisciplinary collaboration to develop integrative care programs for these underserved groups. Methods: Philanthropic funding supported part-time child life specialist and creative arts therapist deployment to develop and implement integrative virtual group programs, as well as interdisciplinary integrative programs, to serve SCYC. Attendance, engagement, and qualitative feedback were used for program iteration and supported the transition to institutional funding. Results: Integrative programs provided 331 caregiver and sibling encounters during the two-year pilot. Qualitative feedback from caregivers highlighted the value of virtual services in reaching geographically dispersed families and addressing feelings of isolation among SCYC at the universal and targeted levels of care. Communication about these key outcomes led to operational funding and sustained integrated care programs. Conclusions: This manuscript illustrates a successful model of leveraging philanthropic funding to support the development of integrative care programs to serve SCYC. Future research should focus on refining the clinical and financial feasibility of such models and assessing their impact on family well-being.

## 1. Introduction

Ten years ago, the Standards for Psychosocial Care for Children with Cancer and their Families were published in a Special Issue of Pediatric Blood and Cancer [1]. Supported by the Mattie Miracle Foundation [2], this set of 15 evidence-based standards was developed by the multidisciplinary Psychosocial Standards of Care Project for Childhood Cancer, which consisted of more than 80 oncology professionals and parent advocates and endorsed by key professional organizations. Each standard includes a rigorous review of the scientific literature in support of providing youth with cancer and their family members with psychosocial care across a range of clinical domains in support of optimal outcomes. Two of these standards emphasize the unique psychosocial needs of siblings [3] and caregivers [4] of youth with cancer (SCYC), as well as the importance of delivering psychosocial support to these family members.

Although largely resilient, caregivers of youth with cancer are profoundly impacted by their child’s cancer diagnosis and treatment [4]. Many caregivers experience elevated levels of distress and some develop clinically significant depressive symptoms, anxiety symptoms, and symptoms of post-traumatic stress at some point during their child’s treatment or even into survivorship [5]. Greater caregiver distress is associated with lower quality of life [6] and greater child distress [1,2] and it can negatively impact self-worth of siblings bereaved by cancer [3].

Siblings of youth with cancer, although similarly resilient, experience a range of psychosocial impacts including emotional distress, separation from their sibling and caregiver, and disruption of school attendance, family routines, and other recreational activities [4,5,6,7]. Despite these impacts, Pariseau and colleagues [8] found that caregivers may be unaware of siblings’ distress due to caregivers’ heightened stress, decreased communication, lower tolerance for siblings’ negative emotions as well as sibling’s reticence to disclose their distress to caregivers in an attempt to align with family messaging (e.g., positivity) and reduced reliance on caregivers for emotional support.

Despite the strong evidence in support of providing SCYC with psychosocial services aimed at alleviating distress and improving resilience, psychosocial care for SCYC remains under implemented [9,10,11,12]. In their implementation assessment, Scialla and colleagues [10] surveyed 144 pediatric oncology treatment programs in the United States and found that although many programs provide some degree of psychosocial support to youth with cancer and their families, barriers to systematic care delivery consistent with the Standards include understaffed psychosocial workforce, pragmatic issues related to funding and reimbursement, and lack of time to provide care. In their follow-up study, Scialla and colleagues [11] found that sibling psychosocial services are among the least implemented standards for psychosocial care.

Jones and colleagues [9] surveyed social workers from 81 treating institutions in the United States and found that barriers to implementing psychosocial care for siblings, in addition to staffing shortages, include distance from the treating institution, logistical barriers faced by caregivers struggling to manage the varied needs of their ill and healthy children, visitor restrictions (e.g., during flu season), transportation expenses, and lack of psychosocial service availability during after-school hours. Brosnan and colleagues [13] described psychosocial care provider’s perceptions of the barriers to implementing standards of care for siblings of youth with cancer. They found that barriers to services exist at multiple levels (healthcare system, treatment center, family) and often interact with each other in varying ways, resulting in limited service availability and limited utilization, which ultimately reinforces deprioritization of funding and decreased delivery of psychosocial services for siblings.

In addition to the implementation challenges described above, the provision of psychosocial care to adult caregivers within a pediatric institution can be further complicated by regulatory and ethical challenges [14]. Caregivers have self-identified prioritizing the needs of their ill child over their own needs, reticence to leave their ill child’s bedside and feeling as if they have sufficient psychosocial support as primary reasons for not participating in psychosocial services [15].

Although systemic barriers to implementation remain daunting and underscore challenges in delivering psychosocial services to SCYC, several efforts have been made to mitigate these barriers. With regard to funding and reimbursement barriers impacting implementation more broadly, Scialla and colleagues [10] recommend engaging stakeholders, including healthcare administrators and parent advocates. These critical collaborators may provide both business and operational partnership as well as values-driven champions for psychosocial services. These partnerships may be necessary to promote operational funding in the absence of reimbursement models and may eventually support outcome driven reimbursement models.

Scialla and colleagues [10] also posited that lack of interdisciplinary teams pose challenges to implementation of the Standards. Although pediatric psychosocial oncology programs may not be best suited to meet acute mental health needs of SCYC, models of care such as the Pediatric Psychosocial Preventative Health Model [16] (PPPHM) describe universal and targeted tiers of care through which an interdisciplinary workforce can perhaps more effectively deliver care consistent with the Standards for SCYC [17] without referring outside of the psychosocial program for support [11]. Notably, pragmatic and reimbursement challenges were identified as primary barriers to funding more robust interdisciplinary teams, which may impact a program’s ability to implement psychosocial programs aligned with the Standards. However, other than describing the number of staff measured in full time equivalent (FTE) of social workers, child life specialists, psychologists, psychiatrists, and neuropsychologists, the authors did not capture the availability of subspecialties that provide integrative care, such as creative arts therapists.

A potential strategy to enhance interdisciplinary teams is by increasing integrative care for SCYC. Integrative care is a model of holistic care intended to relieve suffering and support resilience as youth and their families manage the physical, emotional, social, and spiritual impacts of critical illness [18]. Integrative care interventions have been shown to be an important component of psychosocial care for children with cancer and their families [19,20,21]. Specifically, creative arts therapy groups have improved aspects of psychosocial functioning of siblings of youth with cancer [22,23,24,25] including positive impacts on siblings’ self-esteem, somatic symptoms, and externalizing symptoms [26] and overall benefit to social and emotional health of the ill child’s family members [27]. Mind–body techniques such as yoga, mindfulness, and expressive arts therapies have demonstrated promise in reducing stress and improving quality of life in both caregivers and siblings [20]. These approaches can enhance self-regulation, promote emotional well-being, and provide meaningful outlets for processing complex emotions [28,29,30]. Cancer impacts the entire family system, often altering relationships, routines and roles [31,32]. Family-centered interventions that promote open communication, collaborative problem solving, and mutual support may mitigate familial strain and foster adaptive functioning [33,34,35]. Group activities such as group art projects foster cohesion and provide a positive shared experience, helping siblings and caregivers feel more connected to each other and the child with cancer [36,37].

Similarly to the barriers to psychosocial services described earlier, access barriers to integrative care for SCYC often arises from individual as well as systemic and logistical factors. These include lack of qualified personnel, space constraints, and lack of adequate materials needed for effective implementation of integrative care interventions [10]. Lack of awareness, on behalf of referring providers or of patients and families themselves, of the benefits of integrative care [38] may also limit access to integrative care, even if it is available. Lack of virtual options for delivering integrative care may also limit accessibility for geographically dispersed SCYC. Finally, time constraints and emotional fatigue may also contribute to deprioritization of interventions that are deemed less critical or central to the ill child’s medical recovery [13].

Given the myriad barriers to integrative care for SCYC, our goal was to describe the development and implementation of integrative psychosocial services for these underserved groups. We present a model for leveraging short-term funding opportunities and maximizing interdisciplinary collaboration to develop and deliver integrative care programs for SCYC. We also provide examples of integrative care interventions that were implemented with both siblings as well as caregivers of youth with cancer and describe our strategy for securing operational funding of these services to ensure their sustainability. To guide interpretation and highlight generalizable lessons, we draw on implementation science frameworks to discuss this program development

## 2. Materials and Methods

In an effort to enhance access to integrative psychosocial services for SCYC at a large Children’s Hospital in the Pacific Northwest, we designed a model to leverage short-term funding, optimize interdisciplinary staffing opportunities, engage multiple clinical and administrative partners to ensure sustainability and comprehensive accountability for outcomes, and promote equitable resource allocation. Implementation science constructs, including reach, adoption, implementation process, and maintenance, informed our project design.

### 2.1. Pilot Funding

We identified a short-term funding opportunity through an institutionally affiliated philanthropic organization whose mission aligned with supporting youth with behavioral health needs. Our proposal highlighted the overlapping values of our pediatric oncology psychosocial program, “To develop interdisciplinary and innovative models of psychosocial care with the Cancer and Blood Disorders center to be a leader for equitable delivery of comprehensive psychosocial care to children with cancer and their families” with the vision of the philanthropic organization, “To provide hope, care and cures to help every child live the healthiest and most fulfilling life possible.”

Our proposal included a description of the Standards [39], with specific focus on the psychosocial services that are recommended for SCYC [4,40]. We also described our psychosocial program’s use of the Matrix [41] to evaluate the implementation of each of these standards of care within our program. The Matrix offers a method of scoring the degree to which each standard has been implemented on a 5-point scale with 1 indicating complete lack of or insufficient level of implementation of the standard and 5 indicating comprehensive care consistent with the standard and complete implementation [12].

Sibling Proposal: The funding proposal highlighted that, at baseline, our psychosocial program was unable to consistently provide siblings with assessment of their psychosocial risk, adjustment, or needs, nor were siblings routinely provided with psychosocial services. The proposal included strategies to enhance psychosocial care for siblings including providing individual and group-level integrative interventions to siblings in support of their coping, family relationships, and peer support. Our proposal asserted the expectation that implementation of these strategies would raise the Matrix rating from a baseline of 1.5 to 3 indicating greater adherence to the standard of psychosocial care for siblings of youth with cancer.

Caregiver Proposal: The funding proposal also described the low Matrix implementation rating of caregiver services within our psychosocial program, noting that at baseline, caregivers did not have routine access to psychosocial interventions aimed at supporting caregiver coping at every stage of a child’s pediatric cancer experience. The proposed integrative psychosocial interventions, including offering individual and family-focused services to caregivers, would enhance care consistently with the standard from baseline rating of 1.5 to 3 by ensuring that caregivers have access to psychosocial interventions for their own coping needs.

### 2.2. Interdisciplinary Model of Integrative Care

We designed a model to optimize interdisciplinary staffing in support of enhancing access to integrative psychosocial services for SCYC [10,17] and in support of the pediatric preventative model of care [16]. Child life and creative arts disciplines were selected intentionally due to complementary clinical training and scope of service. Board-certified child life specialists have unique training and expertise in helping children and families understand and cope with medical diagnoses and treatments; facilitate coping and resilience through play, self-expression, and communication; and can provide assessment and recommendations regarding the developmental needs of youth within medical contexts. Creative arts therapists comprise a broad range of disciplines with distinct training and credentials including art therapists, music therapists and expressive arts therapists. Creative arts therapists combine psychological theory and creative art interventions to improve mental and emotional well-being through fostering self-awareness and enhancing coping strategies. Together, these roles were instrumental in designing interventions aimed to enhance coping and well-being of SCYC.

We requested funding to support .2 FTE for a creative arts therapist, .8 FTE for a child life specialist, and .8 FTE for an administrative coordinator role (which was dedicated to these and other psychosocial programs) to support the delivery of integrative care services for SCYC at the universal and targeted tiers. This collaboration contributed to the design of interdisciplinary interventions for SCYC, including the specific integrative care services described in the Section 3. The rationale for this split in FTE for both the Child Life Specialist and the Creative Arts Therapist was pragmatic. Due to the overwhelming demand for child life services within our program, it was not feasible to extend existing child life FTE to serve SCYC. Thus, a new .8 FTE child life position was proposed. A board-certified art therapist working part-time at the institution (in a different clinical area within the Creative Arts service line) had availability to expand her FTE and had an interest in working with families of youth with cancer. Although the person that we hired into this role is an art therapist, we use the term creative arts therapist throughout this manuscript to acknowledge the broad range of non-mental health focused but still therapeutically trained professionals who are well suited to providing integrative care interventions.

### 2.3. Communication

We implemented routine communication about the new integrative psychosocial services for SCYC into regular psychosocial meetings with program staff (psychology, social work, child life, creative arts, palliative care, education services, spiritual care) as recommended by Wiener and colleagues [17]. We engaged our funding partners as well as clinical and administrative leadership from oncology, psychology, creative arts, and child life with routine communication about the services offered through this short-term funding opportunity. These communications were intended to enhance awareness of and increase access to these newly developed services for SCYC and to engage clinical and administrative partners in sharing accountability for sustainability and program outcomes.

### 2.4. Equity

We designed integrative psychosocial services to promote equitable access for SCYC who may face a number of barriers in accessing care. First, we designed integrative care services at the universal and targeted tier levels for SCYC to ensure that the majority of families of youth with cancer at our institution would be eligible and appropriate for services. Second, we designed psychosocial services that would be available via telehealth so that geographically dispersed SCYC could still access services and benefit from social connection and integrative care. Finally, due to our institution’s large catchment area, which includes multiple states, we ensured that integrative services were provided by non-billing, non-credentialed psychosocial providers (child life and creative arts) so that state licensure restrictions did not prevent SCYC residing outside of the state where the child was receiving cancer treatment from accessing integrative services. Our design also ensured that services would be free of charge to all families so that they would be accessible for SCYC regardless of their ability to pay for these services. Interpreter services and tablets were available to all siblings and caregivers as a standard of care within our pediatric oncology psychosocial program.

Our proposal described an intention to trial integrative care programs for SCYC and use attendance, engagement, and qualitative feedback to inform iterative program evaluation and to guide subsequent attempts to procure permanent operational funding following a 2-year pilot period.

## 3. Results

The pilot funding proposal for integrative care for SCYC was funded with .2 FTE for a creative arts therapist, .8 FTE for a child life specialist, and .8 FTE for an administrative coordinator for a two-year pilot period. The administrative coordinator position was intended to support the integrative care services for SCYC as well as other psychosocial services within our pediatric oncology program.

### 3.1. Integrative Care Services for Siblings and Caregivers of Youth with Cancer

Initial funding in 2020 coincided with the COVID-19 pandemic, which impacted our pediatric psychosocial oncology program as a whole and also impacted our hiring timeline and retention for the funded positions due to uncertainty in how psychosocial care would continue to be delivered at our institution. Once fully staffed, interdisciplinary integrative care services were developed in collaboration between child life, creative arts, and psychology with interdisciplinary leadership oversight. The individual and group services for siblings and caregivers are listed in Table 1.

Siblings (and their caregivers) were provided with opportunities to receive support from the Child Life Specialist related to understanding and coping with pediatric cancer and cancer treatment. Siblings were also able to schedule individual creative arts sessions with the Creative Arts Therapist to support coping. Several group interventions were also developed to provide opportunities for social connection, creative arts expression, and coping skill building [42]. Group activities and creative arts interventions and materials were proposed by the Creative Arts Therapist and reviewed by the Child Life Specialist to ensure developmental appropriateness and relevance to pediatric cancer.

*Ink About It* was the first sibling group that was implemented within this program. The group was intended to provide siblings ages 8–13 with opportunities to reflect on their unique sibling experiences with peers likely to have had similar experiences. This group did not include caregivers or patients with cancer in an effort to decrease the likelihood that siblings may inhibit reflecting honestly about their experiences. The eight-week virtual group was facilitated by the Child Life Specialist and Creative Arts Therapist and included therapeutic art activities in support of building emotion identification and coping through creative expression. Mindfulness activities were also facilitated in this group. Due to the positive feedback from this group and specific requests from group members to also include their sibling with cancer in similar integrative care interventions, the *Art and Heart Space* group was developed.

The *Art and Heart Space* group was a six-week virtual group that included patients with cancer and their siblings ages 7–12. This group was also facilitated by the Child Life Specialist and Creative Arts Therapist and included opportunities to reflect on thoughts, feelings and experiences with cancer as well as therapeutic art activities in support of building emotion identification and coping through creative expression.

Positive caregiver feedback and specific requests for groups for teens prompted the development of the *Dessert and Draw* group, which was a virtual group intended to support teen patients with cancer and their teen siblings. Similarly to the other sibling groups, this group was intended to provide opportunity to reflect on the impact of cancer and treatment on patients and siblings, and to use creative arts to identify and express emotions, and to connect with other youth with similar experiences. Specialized art materials including oil pastels and a journal, as well as dessert items were included to incentivize participation and provide positive experiences for group members. Unfortunately, due to low attendance, this group was discontinued after three sessions.

Materials for each of the above groups were compiled and mailed to families in advance of the group. The age range for each of these groups was intentional in an effort to maximize the intended benefits of the group. The younger age limit was determined to ensure that participants were able to self-manage creative arts materials during a 60 min virtual session; engage appropriately with other group members; navigate the virtual technology (e.g., muting, using the chat function); and find benefit from discussing sibling experiences. The older age-limit was set to keep a relatively developmentally consistent experience across attendees. Although the *Ink About It* and *Art and Heart Space* groups ran multiple rounds and were generally well attended, the *Dessert and Draw* group was discontinued due to low attendance.

The Creative Arts Therapist and Child Life Specialist used multiple methods to assess group member engagement and response to the group interventions, including inquiry, discussion, use of the chat tool in the virtual platform, and sometimes asking participants to rate their distress on a scale from 0 to 100 at the beginning and end of the session. These strategies also allowed for follow-up conversations with group members, their caregivers, and sometimes siblings were triaged to higher levels of care as needed.

Caregivers were able to schedule individual creative arts sessions with the Creative Arts Therapist. Caregivers were also able to join creative arts therapy groups facilitated by the Creative Arts Therapist. The *Mindfulness and Collage* caregiver group was developed to provide caregivers with an approachable, low-stakes creative arts activity as well as opportunity to receive and provide support from other caregivers of youth with cancer. The group was facilitated by the Creative Arts Therapist, who also led mindfulness activities to support coping. This group was scheduled monthly and sometimes conducted virtually and sometimes organized to occur in person with caregivers of admitted children. The in-person groups were better attended, perhaps due to inpatient youth having attentive care by bedside nursing staff. Collage materials were mailed to caregivers in advance of virtual groups or were delivered to caregivers of children during inpatient admissions or clinic visits.

The integrative psychosocial services for SCYC described above resulted in 331 caregiver and sibling encounters, which were primarily delivered via telehealth. By the end of the two-year pilot period, individual sibling encounters averaged 18 per month and individual caregiver encounters averaged 9 per month. Qualitative feedback from caregivers included spontaneous communication to staff, highlighting the value of virtual services in reaching geographically dispersed families and addressing feelings of isolation among siblings at the universal and targeted levels of care:

“We have appreciated these virtual art therapy sessions for both siblings. Coming off of treatment and into pandemic has been difficult to regain a semblance of normalcy. And trying to find friends beyond a quick clinic visit was challenging. But my kids have gained not just a time to speak to others, gain confidence but also to make friendships. Thank you for providing a fun and safe space for the kids to learn and grow.”

Qualitative feedback from siblings also highlighted the importance of integrative care in supporting sibling coping:

“The whole time making artwork has been personal. Everything I had gone through watching my sibling have cancer had been hard to process. But this has been a great time just to express on paper or drawings what it has been like for me.”

### 3.2. Operational Funding

There was precedent at our institution for successful pilot programs (revenue generating, non-revenue generating, and programs operating at a loss) to be sustained with operational, grant, or philanthropic funding after initial pilot funding expired. Funding sustainability was not considered to be a major risk when we initially applied for the pilot funding. However, we did not anticipate the COVID-19 pandemic, which coincided with our initial funding, or the financial impact that it had on healthcare and healthcare funding in the years that followed.

In 2023, the healthcare sector, particularly hospitals, experienced a notable increase in layoffs compared to previous years [43]. This trend was attributed to several factors, including financial challenges, rising operational costs, and workforce shortages [44]. Despite these national trends, which also impacted our institution, we were able to successfully convert the Child Life Specialist and the Creative Arts Therapist FTE to operational funding in 2023 when our proposal was approved as part of annual operational budgeting.

Our efforts to secure this funding included six months of preparation by the Child Life Specialist, the Creative Arts Therapist and psychology and child life leadership. We drafted a proposal using a standardized institutional format and detailed the above results of this pilot, including the number of sibling and caregiver encounters, a description of the individual and group programming, as well as quotes from siblings and caregivers. This data demonstrated not only the pragmatic viability of these integrative, interdisciplinary care services, but the value of these programs to our patients and families consistent with the family-centered mission of the institution.

We explicitly described lack of alternative options for program sustainability if the direct care provider positions were not included in the institution’s annual operational budget. We engaged operational and financial managers across three divisions (Psychiatry, Child Life, and Oncology) in alignment meetings in advance of budget review season where explicit support for program sustainability was expressed across leaders. Finally, we aligned on a request to Oncology senior leadership to include the funding proposal for the Creative Arts Therapist, the Child Life Specialist, and the Administrative Coordinator FTE within the Oncology annual operational budget. The Oncology division was the only one of these three divisions with a positive operating margin.

Although our Child Life Specialist and Creative Arts Therapist FTE were approved by senior operational and financial leadership and added to the annual operational budget, we were unable to procure operational funding for the Administrative Coordinator that supported integrative care and other pediatric oncology psychosocial programs. During the pilot funding phase, the Administrative Coordinator provided substantial support for group recruitment (passing out flyers), collating and mailing group materials, room reservations for in-person groups, and maintaining family-facing group materials. When the role was eliminated due to lack of operational funding, these tasks fell to the Child Life Specialist and the Creative Arts Therapist, decreasing the time that they were able to provide direct care to SCYC due to lack of alternative administrative support within the psychosocial program.

## 4. Discussion

This manuscript describes a successful model for leveraging short-term philanthropic funding and interdisciplinary collaboration to implement integrative psychosocial services for SCYC. This initiative demonstrated the viability of creating and delivering integrative care for SCYC by maximizing interdisciplinary expertise and collaboration. The creative arts and child life interventions, which were delivered both individually and in groups, largely via telehealth and regardless of geographical location, enhanced our ability to deliver care consistent with the Standards to SCYC. The transition of the Child Life Specialist and Creative Arts Therapist positions from pilot funding to operational support further illustrates the potential for engaging short-term funding to integrate such services into institutional care models, even amidst a shifting healthcare landscape.

To better situate this program within the broader implementation science literature, the development and evaluation of our pilot can be understood through the lens of the Exploration, Preparation, Implementation, Sustainment (EPIS) framework [45]. In the Exploration phase, program leaders identified a gap in psychosocial services for siblings and caregivers, particularly in addressing isolation, emotional distress, and resilience. The Preparation phase was supported by philanthropic funding and interdisciplinary collaboration, which enabled pragmatic staffing models, creative use of telehealth, and iterative program design with input from caregivers and interdisciplinary professionals. The Implementation phase emphasized integrative, multimodal care, including virtual groups, mailed expressive arts materials, and attention to reducing access barriers to promote equitable care. Finally, the Sustainment phase was marked by the transition from pilot to operational funding, reflecting institutional adoption of the program, though ongoing challenges in administrative support underscore the difficulty of maintaining novel psychosocial interventions within complex medical systems.

In parallel, outcomes can be interpreted through the RE-AIM framework [46]. The program demonstrated meaningful Reach, with 331 encounters across siblings and caregivers, and Effectiveness, evidenced by participant feedback indicating improved coping, reduced isolation, and strengthened family resilience. Adoption was reflected in broad buy-in from interdisciplinary teams and philanthropic partners, while Implementation strategies included leveraging telehealth, mailed resources, and pragmatic staffing to maximize feasibility. Partial transition to institutional support highlights early progress toward Maintenance, while also identifying areas for improvement in sustaining administrative infrastructure.

By applying these frameworks, this pilot contributes generalizable insights into how pediatric oncology programs can design, implement, and sustain integrative psychosocial care for SCYC. These findings may inform future efforts to scale integrative psychosocial interventions and to systematically evaluate outcomes related not only to clinical benefit but also to long-term feasibility and sustainability.

Although encouraging, this pilot project had several limitations, which warrant further discussion. First, this was not a formal research study, and we did not prospectively collect quantitative data to assess outcomes such as coping, family functioning, or satisfaction. Similarly, as this effort was not formally evaluated through a quality improvement initiative or structure, we were not able to analyze the feasibility of these services for SCYC within our institution. We also did not systematically gather demographic information of SCYC who participated in these services, nor of SCYC who did not participate in these services, which limits our understanding of potential barriers to this care.

Second, the program was developed and implemented during the COVID-19 pandemic, which was a period marked by rapid changes in telehealth policy, caregiver availability, and healthcare staffing. It is therefore unclear whether similar implementation and engagement outcomes would have occurred under different conditions. Finally, although qualitative feedback from families was overwhelmingly positive, we did not formally evaluate the feasibility, acceptability, or psychosocial impact of the integrative care services provided.

Future research should focus on rigorous evaluation of both clinical and financial feasibility of integrative psychosocial models for SCYC. Studies assessing program reach, equity, and outcomes such as emotional well-being, coping, and family resilience are essential to inform broader implementation. Additionally, efforts to explore sustainable funding mechanisms such as hospital operations, reimbursement models, or value-based care frameworks will be critical to ensure long-term accessibility of these services. By addressing these next steps, future programs can continue to build on this model to meet the complex, often unmet, psychosocial needs of siblings and caregivers of youth with cancer.

## Figures and Tables

**Table 1:** Integrative care interventions for siblings and caregivers of youth with cancer.

Intervention	Modality	Population	Description
Therapeutic Art Services ^1^	Individual	Siblings (all ages)	Individual sessions available upon referral or request.
Sibling Support ^2^	Individual or family	Siblings (all ages) or family	Individual interventions for siblings, or caregivers of siblings, to provide education and coping support. Available upon referral or request.
Art and Heart Space Group ^1,2^	Group	Patients and Siblings (ages 7–12)	Weekly, 6-session virtual support group with themed creative arts interventions to support coping, increase socialization, and reduce isolation.
Ink About It ^1,2^	Group	Siblings (ages 8–13)	Weekly, 8-session virtual support group with themed creative arts interventions to support coping and adjustment.
Dessert and Draw ^1,2^	Group	Patients and Siblings (teen)	Monthly, drop-in, virtual support group with themed creative arts interventions to support sibling relationship, support coping, and facilitate adjustment.
Mindfulness and Collage ^1^	Group	Caregivers	Bi-weekly, drop-in, in-person group for caregivers of inpatient youth with cancer.
Therapeutic Art Services ^1^	Individual	Caregivers	Individual sessions available upon referral or request.

^1^ Delivered by the Creative Arts Therapist ^2^ Delivered by the Child Life Specialist.

## Data Availability

Not applicable.

## Abbreviations

The following abbreviations are used in this manuscript:

SCYC	Siblings and caregivers of youth with cancer
PPPHM	Pediatric Psychosocial Preventative Health Model
FTE	Full-Time Equivalent

## References

[1] Bakula D.M., Sharkey C.M., Perez M.N., Espeleta H.C., Gamwell K.L., Baudino M., Delozier A.M., Chaney J.M., Alderson R.M., Mullins L.L. (2019). Featured Article: The Relationship Between Parent and Child Distress in Pediatric Cancer: A Meta-Analysis. J. Pediatr. Psychol..

[2] Trask P.C., Paterson A.G., Trask C.L., Bares C.B., Birt J., Maan C. (2003). Parent and Adolescent Adjustment to Pediatric Cancer: Associations with Coping, Social Support, and Family Function. J. Pediatr. Oncol. Nurs..

[3] Garcia D., Olsavsky A.L., Hill K.N., Patterson V., Baughcum A.E., Long K.A., Barrera M., Gilmer M.J., Fairclough D.L., Akard T.F. (2023). Associations between Parental Depression, Communication, and Self-Worth of Siblings Bereaved by Cancer. J. Fam. Psychol..

[4] Gerhardt C.A., Lehmann V., Long K.A., Alderfer M.A. (2015). Supporting Siblings as a Standard of Care in Pediatric Oncology. Pediatr. Blood Cancer.

[5] Wilkins K.L., Woodgate R.L. (2005). A Review of Qualitative Research on the Childhood Cancer Experience From the Perspective of Siblings: A Need to Give Them a Voice. J. Pediatr. Oncol. Nurs..

[6] Weiner J.A., Woodley L.K. (2018). An Integrative Review of Sibling Responses to Childhood Cancer. J. Child Adolesc. Psychiatr. Nurs..

[7] Long K.A., Lehmann V., Gerhardt C.A., Carpenter A.L., Marsland A.L., Alderfer M.A. (2018). Psychosocial Functioning and Risk Factors among Siblings of Children with Cancer: An Updated Systematic Review. Psychooncology.

[8] Pariseau E.M., Chevalier L., Muriel A.C., Long K.A. (2020). Parental Awareness of Sibling Adjustment: Perspectives of Parents and Siblings of Children with Cancer. J. Fam. Psychol..

[9] Jones B., Currin-Mcculloch J., Pelletier W., Sardi-Brown V., Brown P., Wiener L. (2018). Psychosocial Standards of Care for Children with Cancer and Their Families: A National Survey of Pediatric Oncology Social Workers. Soc. Work Health Care.

[10] Scialla M.A., Canter K.S., Chen F.F., Kolb E.A., Sandler E., Wiener L., Kazak A.E. (2017). Implementing the Psychosocial Standards in Pediatric Cancer: Current Staffing and Services Available. Pediatr. Blood Cancer.

[11] Scialla M.A., Canter K.S., Chen F.F., Kolb E.A., Sandler E., Wiener L., Kazak A.E. (2018). Delivery of Care Consistent with the Psychosocial Standards in Pediatric Cancer: Current Practices in the United States. Pediatr. Blood Cancer.

[12] Wiener L., Canter K., Long K., Psihogios A.M., Thompson A.L. (2020). Pediatric Psychosocial Standards of Care in Action: Research That Bridges the Gap from Need to Implementation. Psychooncology.

[13] Brosnan P., Davis K.A., Mazzenga M., Oberoi A.R., Sharkey C.M., Buchbinder D., Alderfer M.A., Long K.A. (2022). Psychosocial Care Providers’ Perspectives: Barriers to Implementing Services for Siblings of Children with Cancer. Pediatr. Blood Cancer.

[14] Patten J.T., Hoag J.A., Galtieri L.R., Canavera K., Thompson A.L. (2022). Suicidality in Bereaved Parents Within Pediatric Institutions: Recommendations for Managing Ethical Challenges. Clin. Pract. Pediatr. Psychol..

[15] Devine K.A., Manne S.L., Mee L., Bartell A.S., Sands S.A., Myers-Virtue S., Ohman-Strickland P. (2016). Barriers to Psychological Care among Primary Caregivers of Children Undergoing Hematopoietic Stem Cell Transplantation. Support Care Cancer.

[16] Kazak A.E. (2006). Pediatric Psychosocial Preventative Health Model (PPPHM): Research, Practice, and Collaboration in Pediatric Family Systems Medicine. Fam. Syst. Health.

[17] Wiener L., Kazak A.E., Noll R.B., Patenaude A.F., Kupst M.J. (2015). Interdisciplinary Collaboration in Standards of Psychosocial Care. Pediatr. Blood Cancer.

[18] Lee J., Kim M.S., Shin H.Y. (2020). Integrative Care for Children with Medical Complexity. Clin. Exp. Pediatr..

[19] Knott D., Krater C., MacLean J., Robertson K., Stegenga K., Robb S.L. (2022). Music Therapy for Children with Oncology & Hematological Conditions and Their Families: Advancing the Standards of Psychosocial Care. J. Pediatr. Hematol. Nurs..

[20] Stritter W., Everding J., Luchte J., Eggert A., Seifert G. (2021). Yoga, Meditation and Mindfulness in Pediatric Oncology—A Review of Literature. Complement. Ther. Med..

[21] Yousefi F., Moradveisi B., Roshani D., Mansouri M., Servatyari K. (2024). Hypnotherapy, Relaxation, and Music Therapy in Pediatric Cancer Pain Management: A Clinical Trial Comparison. Open Pain J..

[22] Barrera M., Chung J.Y.Y., Greenberg M., Fleming C. (2002). Preliminary Investigation of a Group Intervention for Siblings of Pediatric Cancer Patients. Child. Health Care.

[23] Barrera M.E., Rykov M.H., Doyle S.L. (2002). The Effects of Interactive Music Therapy on Hospitalized Children with Cancer: A Pilot Study. Psychooncology.

[24] Dolgin M.J., Somer E., Zaidel N., Zaizov R. (1997). A Structured Group Intervention for Siblings of Children with Cancer. J. Child Adolesc. Group Ther..

[25] Nolbris M., Abrahamsson J., Hellstrom A.-L., Olofsson L. (2010). The Experience of Therapeutic Support Groups by Siblings of Children with Cancer. Pediatr. Nurs..

[26] Jo M.-J., Hong S., Park H.R. (2018). Effects of Art Intervention Program for Siblings of Children with Cancer: A Pilot Study. J. Pediatr. Oncol. Nurs..

[27] Bally J.M.G., Burles M., Spurr S., McGrath J. (2023). Exploring the Use of Arts-Based Interventions and Research Methods in Families of Seriously Ill Children: A Scoping Review. J. Fam. Nurs..

[28] Bockmann J.O., Yu S.Y. (2023). Using Mindfulness-Based Interventions to Support Self-Regulation in Young Children: A Review of the Literature. Early Child. Educ. J..

[29] Farris S.R., Grazzi L., Holley M., Dorsett A., Xing K., Pierce C.R., Estave P.M., O’Connell N., Wells R.E. (2021). Online Mindfulness May Target Psychological Distress and Mental Health during COVID-19. Glob. Adv. Health Med..

[30] Zhu L., Li L., Li X., Wang L. (2022). Mind–Body Exercises for PTSD Symptoms, Depression, and Anxiety in Patients With PTSD: A Systematic Review and Meta-Analysis. Front. Psychol..

[31] Bates C.R., Pallotto I.K., Moore R.M., Fornander M.J., Covitz L.M., Dreyer Gillette M.L. (2021). Family Rules, Routines, and Caregiver Distress during the First Year of Pediatric Cancer Treatment. Psychooncology.

[32] Santos S., Crespo C., Canavarro M.C., Kazak A.E. (2015). Family Rituals and Quality of Life in Children With Cancer and Their Parents: The Role of Family Cohesion and Hope. J. Pediatr. Psychol..

[33] Koumarianou A., Symeonidi A.E., Kattamis A., Linardatou K., Chrousos G.P., Darviri C. (2021). A Review of Psychosocial Interventions Targeting Families of Children with Cancer. Palliat. Support. Care.

[34] Peikert M.L., Inhestern L., Bergelt C. (2018). Psychosocial Interventions for Rehabilitation and Reintegration into Daily Life of Pediatric Cancer Survivors and Their Families: A Systematic Review. PLoS ONE.

[35] Aguilar B.A. (2017). The Efficacy of Art Therapy in Pediatric Oncology Patients: An Integrative Literature Review. J. Pediatr. Nurs..

[36] Guan T., Chapman M.V., Qan’ir Y., Song L. (2021). Psychosocial Interventions for Siblings of Children with Cancer: A Mixed Methods Systematic Review. Psychooncology.

[37] Barrera M., Neville A., Purdon L., Hancock K. (2018). “It’s Just for Us!” Perceived Benefits of Participation in a Group Intervention for Siblings of Children with Cancer. J. Pediatr. Psychol..

[38] Womack D.M., Kennedy R., Chamberlin S.R., Rademacher A.L., Sliney C.D. (2022). Patients’ Lived Experiences and Recommendations for Enhanced Awareness and Use of Integrative Oncology Services in Cancer Care. Patient Educ. Couns..

[39] Wiener L., Kazak A.E., Noll R.B., Patenaude A.F., Kupst M.J. (2015). Standards for the Psychosocial Care of Children with Cancer and Their Families: An Introduction to the Special Issue. Pediatr. Blood Cancer.

[40] Kearney J.A., Salley C.G., Muriel A.C. (2015). Standards of Psychosocial Care for Parents of Children with Cancer. Pediatr. Blood Cancer.

[41] Wiener L., Kupst M.J., Pelletier W., Kazak A.E., Thompson A.L. (2020). Tools to Guide the Identification and Implementation of Care Consistent with the Psychosocial Standards of Care. Pediatr. Blood Cancer.

[42] Joschko R., Klatte C., Grabowska W.A., Roll S., Berghöfer A., Willich S.N. (2024). Active Visual Art Therapy and Health Outcomes: A Systematic Review and Meta-Analysis. JAMA Netw. Open.

[43] Huang V., Cheng E.P. Job Openings and Hires Decline in 2023 as the Labor Market Cools: Monthly Labor Review: U.S. Bureau of Labor Statistics. Bureau of Labor Statistics. https://www.bls.gov/opub/mlr/2024/article/job-openings-and-hires-decline-in-2023.htm.

[44] Muoio D. 100 Hospital and Health System Layoffs from 2023. https://www.fiercehealthcare.com/providers/layoffs-ramping-among-hospitals-and-health-systems-heres-34-examples-2023.

[45] Aarons G.A., Hurlburt M., Horwitz S.M. (2011). Advancing a Conceptual Model of Evidence-Based Practice Implementation in Public Service Sectors. Adm. Policy Ment. Health Ment. Health Serv. Res..

[46] Glasgow R.E., Vogt T.M., Boles S.M. (1999). Evaluating the Public Health Impact of Health Promotion Interventions: The RE-AIM Framework. Am. J. Public Health.

